# Insulin therapy and blood glucose management in critically ill patients: a 1-day cross-sectional observational study in 69 French intensive care units

**DOI:** 10.1186/s13613-023-01142-9

**Published:** 2023-06-17

**Authors:** Maxime Desgrouas, Julien Demiselle, Laure Stiel, Vincent Brunot, Rémy Marnai, Sacha Sarfati, Maud Fiancette, Fabien Lambiotte, Arnaud W. Thille, Maxime Leloup, Sébastien Clerc, Pascal Beuret, Anne-Astrid Bourion, Johan Daum, Rémi Malhomme, Ramin Ravan, Bertrand Sauneuf, Jean-Philippe Rigaud, Pierre-François Dequin, Thierry Boulain

**Affiliations:** 1 Médecine Intensive Réanimation, Centre Hospitalier Régional d’Orléans, 45100 Orléans, France; 2 Service de Médecine Intensive-Réanimation, Nouvel Hôpital Civil, Hôpitaux Universitaires de Strasbourg, Strasbourg, France; 3 UMR 1260 Nanomedicine Regenerative, INSERM, Université de Strasbourg, Strasbourg, France; 4 Réanimation Médicale, Groupe Hospitalier de la Région Mulhouse Sud Alsace, Mulhouse, France; 5 UMR 1231, Inserm, LNC, Dijon, France; 6 LipSTIC, LabEx, Dijon, France; 7 Médecine Intensive Réanimation, Hôpital Universitaire Lapeyronie, Université de Montpellier, Montpellier, France; 8 Service de Réanimation Médico-Chirurgicale, Centre Hospitalier Le Mans, 72000 Le Mans, France; 9 Medical Intensive Care Unit, Normandie Univ, UNIROUEN, UR 3830, CHU Rouen, 76000 Rouen, France; 10 Service de Médecine Intensive Réanimation, CHD Vendée la Roche Sur Yon, La Roche Sur Yon, France; 11 Service de Réanimation Polyvalente, Centre Hospitalier de Valenciennes, Valenciennes, France; 12 CHU de Poitiers, Médecine Intensive Réanimation, Poitiers, France; 13 Service de Réanimation, Groupe Hospitalier La Rochelle Ré Aunis, La Rochelle, France; 14 Service de Médecine Intensive Et Réanimation (Département R3S), AP-HP, Groupe Hospitalier Universitaire APHP-Sorbonne Université, Site Pitié-Salpêtrière, 75013 Paris, France; 15 Réanimation Et Soins Continus, Centre Hospitalier de Roanne, Roanne, France; 16 Réanimation, Centre Hospitalier de Cholet, Cholet, France; 17 Médecine Intensive Réanimation, Centre Hospitalier Intercommunal Ballanger, Aulnay Sous Bois, France; 18 Service de Réanimation, Centre Hospitalier Antibes Juan-Les-Pins, Antibes, France; 19 Réanimation Polyvalente et Surveillance Continue, Centre Hospitalier de Vichy, Vichy, France; 20 Médecine Intensive Réanimation, Centre Hospitalier Public du Cotentin, 50100 Cherbourg en Cotentin, France; 21 Médecine Intensive Réanimation, Centre Hospitalier de Dieppe, Avenue Pasteur, 76200 Dieppe, France; 22 Médecine Intensive - Réanimation, Hôpital Bretonneau, Tours, France; 23 Centre d’Étude Des Pathologies Respiratoires, UMR 1100, INSERM, Université de Tours, Tours, France; 24 INSERM CIC 1415, Tours, France; 25 CRICS-TriGGERSep Network, Paris, France

**Keywords:** Insulin, Critical care, Blood glucose, Glycaemic control, Hyperglycaemia, Hypoglycaemia

## Abstract

**Background:**

Hyperglycaemia is common in critically ill patients, but blood glucose and insulin management may differ widely among intensive care units (ICUs). We aimed to describe insulin use practices and the resulting glycaemic control in French ICUs. We conducted a multicentre 1-day observational study on November 23, 2021, in 69 French ICUs. Adult patients hospitalized for an acute organ failure, severe infection or post-operative care were included. Data were recorded from midnight to 11:59 p.m. the day of the study by 4-h periods.

**Results:**

Two ICUs declared to have no insulin protocol. There was a wide disparity in blood glucose targets between ICUs with 35 different target ranges recorded. In 893 included patients we collected 4823 blood glucose values whose distribution varied significantly across ICUs (*P* < 0.0001). We observed 1135 hyperglycaemias (> 1.8 g/L) in 402 (45.0%) patients, 35 hypoglycaemias (≤ 0.7 g/L) in 26 (2.9%) patients, and one instance of severe hypoglycaemia (≤ 0.4 g/L). Four hundred eight (45.7%) patients received either IV insulin (255 [62.5%]), subcutaneous (SC) insulin (126 [30.9%]), or both (27 [6.6%]). Among patients under protocolized intravenous (IV) insulin, 767/1681 (45.6%) of glycaemias were above the target range. Among patients receiving insulin, short- and long-acting SC insulin use were associated with higher counts of hyperglycaemias as assessed by multivariable negative binomial regression adjusted for the propensity to receive SC insulin: incidence rate ratio of 3.45 (95% confidence interval [CI] 2.97–4.00) (*P* < 0.0001) and 3.58 (95% CI 2.84–4.52) (*P* < 0.0001), respectively.

**Conclusions:**

Practices regarding blood glucose management varied widely among French ICUs. Administration of short or long-acting SC insulin was not unusual and associated with more frequent hyperglycaemia. The protocolized insulin algorithms used failed to prevent hyperglycaemic events.

**Graphical Abstract:**

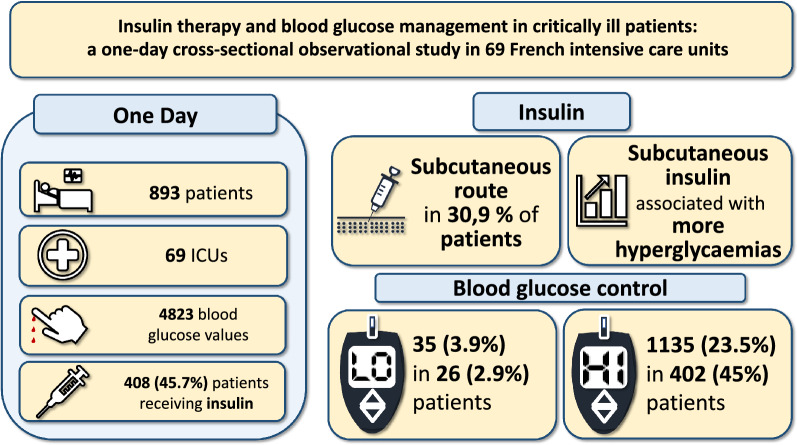

**Supplementary Information:**

The online version contains supplementary material available at 10.1186/s13613-023-01142-9.

## Background

Hyperglycaemia is common in critically ill patients due to increased hepatic output and decreased peripheral glucose uptake as a result of stress, inflammation and the associated decreased insulin sensitivity combined with elevated production of counter-regulatory hormones, such as glucagon or steroids [1]. Hyperglycaemia over 2 g/L was reported to be associated with an increased mortality (Odds-ratio (OR): 2.13 [95% confidence interval (95%CI) 2.03;2.25]) [2]. An early correction of hyperglycaemia with insulin infusion was associated with lower mortality as compared with a delayed management of blood glucose level [3]. In counterpart, severe hypoglycaemias were associated with an increased mortality (OR 2.28 [95% CI 1.41;3.70] in 5365 critically ill patients [4]. In the NICE–SUGAR trial, patients who underwent intensive insulin therapy for tight glucose control had more frequent severe hypoglycaemias than patients in the control arm (6.8% vs 0.5%) and a significantly increased mortality (OR 1.14 [95% CI 1.02–1.28]) [5].

French and international guidelines recommend monitoring the blood glucose level every 2 h in critically ill patients and to address hyperglycaemia with continuous intravenous (IV) insulin infusion. A blood glucose level ≥ 1.5 g/L should trigger the intervention to maintain blood glucose ≤ 1.8 g/L [6-8]. Subcutaneous (SC) short-acting insulin infusion is not recommended before acute phase of organ failures has resolved. Guidelines’ authors positioned for the use of SC insulin only as a relay, considering previous needs in IV insulin and nutritional intakes to calculate the dosing requirement.

However, the way blood glucose is monitored and managed in practice can greatly vary across ICUs [9]. Some authors acknowledged that long-acting SC insulin could be safely used in intensive care patients [10-14]. Others suggested that SC injection of short-acting insulin is safe and effective to achieve targeted blood glucose levels [15]. SC insulin was sometime put forward as a way to decrease workload of nurses, allowing less blood glucose measures and lighter insulin management [16].

Guidelines recommend blood glucose level targets for intensive care patients, but the way to achieve them and the IV delivery route lack robust data. As guidelines state against tight glycaemic control because of an increased risk of hypoglycaemia and mortality, we hypothesized that physicians are thus trying their best to avoid hypoglycaemia, at the cost of a less well-controlled upper limit of blood glucose, and that for that purpose, SC insulin use may not be so unusual [17].

In this context, we undertook an observational study in French ICUs to describe insulin use practices and the resulting glycaemic control according to patients’ characteristics and the phase of the acute illness (i.e., early days of acute organ dysfunction or after) during which patients were observed.

## Methods

In September 2021, we sent an e-mail invitation to participate to 219 intensive care units (ICUs), all members of the CRICS–TRIGGERSEP clinical research network (https://www.crics-triggersep.org/en) and/or of the French Society of Anaesthesia and Intensive Care (SFAR) or French Intensive Care Society (SRLF). A second and third rounds of invitation were sent to non-responders 1 and 2 months after the first e-mail. This multicentre 1-day observational study was planned to be set on November 23, 2021. The participating ICUs received the case report form (CRF) 5 days before this date. Every patient over 18 years of age hospitalized in the participating ICUs on the study day for an acute organ failure, a severe infection or post-operative care was included. Patients were not included if they were admitted in intensive care for ketoacidosis, diabetic coma, severe hypoglycaemia or insulin or oral antidiabetic drugs intoxication. Data were recorded from midnight to 11:59 p.m. the day of the study by 4-h periods. The study was approved by the Ethic Committee of the French Intensive Care Society on 8^th^ August 2021 (CE SRLF 21–67). Patients, or their family, were informed of the study by the Welcome Booklet of each ICU or a dedicated information letter, and their right to refuse participation and how to do so was clearly stated. The study complied with French law and health data protection regulations.

Variables collected included patients’ baseline clinical characteristics, reason for ICU admission, chronic illnesses, chronic medications, insulin type and dose administered on the study day, dietary intakes, sequential organ failure assessment (SOFA) score on the study day [18], number of blood glucose tests performed on the study day, and minimal and maximal values of blood glucose recorded during each 4-h time slot. Blood glucose ranges were determined according to the Society of Critical Care Medicine recommendations [6]. Hypoglycaemia was defined as a blood glucose ≤ 0.7 g/L, severe hypoglycaemia as a blood glucose ≤ 0.4 g/L. Stress hyperglycaemia was defined by a blood glucose ≥ 1.4 g/L. However, as guidelines suggest a moderate rather than tight glucose control range, and recommend treating hyperglycaemia only if ≥ 1.5 g/L to avoid glycaemia ≥ 1.8 g/L, we chose to define hyperglycaemia as a blood glucose ≥ 1.8 g/L.

The individual glucose variability on the study day, i.e., the intra-patient coefficient of variation (standard deviation of blood glucose/mean of blood glucose) [19] was calculated and expressed in percentage in patients with at least two blood glucose measurements within the 24 h of the survey.

As hyperglycaemic stress may vary during an ICU stay, we categorized patients according to the time they have been hospitalized in the ICU when monitored on the study day: those considered to be in the acute, catabolic phase, i.e., when the metabolic disturbances are maximal, and those considered to be in the post-acute phase made of either continuing metabolic instability or anabolism appearance [20]. As the transition time between these two phases is not easily distinguishable for a given patient, we used three different times to separate patients based on that proposed by the European Society for Clinical Nutrition and Metabolism [20]: day 1 vs after day 1, before or after day 3 (≤ / > day 3), and before or after day 7 (< / ≥ day 7). Those three different separation times were tested in our analyses (see below).

### Statistical analysis

We calculated that 400 patients would be enough to estimate the percentage of each categorical binary variable with a confidence interval between −5% and + 5%.

Categorical variables are expressed as numbers and percentages. Continuous variables are expressed as mean and standard deviation (SD) or median and interquartile range (IQR, i.e., 25th and 75th percentiles) depending on their normal or nonnormal distribution as assessed by density and quantile–quantile plots inspection.

Fisher exact test, χ^2^ test, *G* test, *t* test, *F* test, Mann–Whitney *U* test, Kruskal–Wallis test, and Spearman correlation were used for between-group comparisons as appropriate. Median differences and their 95%CI were estimated by bootstrapping (2000 bootstrap samples).

Individual glucose variability on the study day is presented by group as the median value and IQR, as well as its distribution into different classes, i.e., < 15%, 15–30%, and > 30%, the two latter classes being known to be independently associated with higher in-hospital mortality [21].

We used linear mixed modelling with the glycaemia observed in each 4-h period of the study day as the dependent variable to assess the association of the blood glucose level with the following fixed effect covariables: known diabetes, SOFA score measured on the study day, insulin use, ongoing infection, calories intake on the study day (via enteral and parenteral route) per kg of patient’s body weight, oral alimentation or not, 4 h-time slot of the study day during which the glycaemia was measured, and the period of the ICU stay during which each patient was monitored. The aforementioned three ways of dichotomizing the study population according to the observation period were tested. We assumed that ICUs had random intercepts. The association of insulin type and route of administration with the blood glucose level was assessed in the subset of patients receiving insulin on the study day. As some patient’s characteristics may at the same time favour the use of SC insulin and be associated with higher glycaemia (diabetes and previous regular treatment by insulin, for example), the analysis of the association of SC insulin, either of any type or long-acting, with the blood glucose level in the population restricted to patients receiving insulin, was adjusted for confounding using stabilized propensity score-based inverse probability weighing (IPTW) (see Additional file 1 for detailed methods).

We assessed the association of the above-cited covariables with the number of hyperglycaemic (blood glucose level > 1.80 g/L) events per patient using Fisher exact tests and by multivariable mixed effect negative binomial regression. The association of the use of SC insulin with the number of hyperglycaemic events in the subset of patients receiving insulin, was assessed by multivariable mixed effect negative binomial regression with propensity-based IPTW. The ICU was entered as a variable with random intercept. Between-group incidence rate ratios (IRRs) are given with their 95%CI.

For both the linear mixed regression and the negative binomial regression analyses performed on the whole study population, we tested interactions between the variables “diabetes”, “insulin use”, and the “period of observation”. The variables “diabetes”, “IV insulin use”, “SC insulin use” were always kept in the model. We tested all combinations of the remaining variables (SC long-acting insulin use, SOFA score, existence of infection, calories intake, oral alimentation, and time slots of observation) and retained the model with the best fit as selected by the likelihood ratio test.

Analyses were conducted using R 4.0.2 (R Foundation for Statistical Computing). A two-tailed *P* value < 0.05 was considered significant. *P* values for between-group comparisons in the framework of linear mixed models were adjusted for multiple testing by the Tukey method.

## Results

### ICUs’ characteristics

A total of 69 ICUs accepted to participate. The geographical location of each participating ICU is shown in Additional file 1: Fig. S1. Numbers of patients included in each ICU are detailed in Additional file 1: Table S1.

Forty (58.8%) ICUs were mixed (438/893 patients [49%]), 23 (33.8%) medical (396/893 patients [44.3%]), and 5 (7.4%) were other ICUs (59/893 patients [6.6%]) (See Additional file 1: Table S2).

**Fig. 1:**
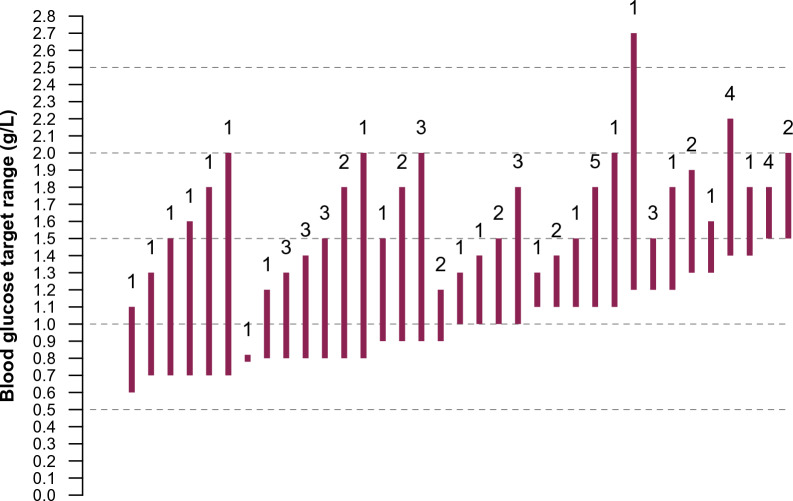
Distribution of blood target ranges in intensive care units. Each vertical bar represents a unique blood glucose target range, extending from the lower to the higher blood glucose target value. Figures above the bars indicate the numbers of centres that used a particular target range. Centres are ranked according to the lower boundary used and then according to the higher boundary used. Note that centres could measure blood glucose in g/L or in mmol/L. In the latter case, mmol/L were converted to g/L and rounded to the nearest decimal place. One centre (seventh target range from the left) had a single blood glucose target (0.8 g/L), which required stopping insulin treatment whenever blood glucose fell below this value, and conversely restarting insulin whenever blood glucose rose above 0.8 g/L. Only one centre used the same narrow target range (0.8–1.1 g/L) as the one originally used by Van der Berghe et al. in 2001 for tight glucose control [33]. The lower boundary of target range used was never as high as the one used by Van der Berghe et al. (1.8 g/L) for patients of the conventional treatment group targeting blood glucose between 1.8 and 2.0 g/L

All ICUs but two (2.9%, representing 17/893 [1.9%] patients) declared having a written service protocol for blood glucose management and insulin administration. Of these, 64 (95.5%) centres provided their insulin protocol in full details. As shown in Fig. 1 there was a wide disparity in blood glucose targets between centres. The lower limit ranged from 0.6 g/L to 1.5 g/L. The upper limit ranged from 0.8 g/L to 2.7 g/L. Thirty-five different target ranges were used. The most used high target value was 1.8 g/L for 27 of the 64 (42.2%) centres that responded to this specific question. The most used low target values were 0.8 g/L for 14 (21.9%) centres and 1.1 g/L for 10 (15.6%) centres.

There was also a wide disparity regarding blood glucose values that either trigger stopping, starting or resuming insulin therapy, as illustrated in Additional file 1: Fig. S2. The standard interval, used first, as well as the shortest interval (depending on blood glucose value or variability) between blood glucose tests performed in patients receiving IV insulin, as set by insulin protocols, also varied widely between centres (Additional file 1: Fig. S3). Some centres never monitor blood glucose more frequently than every 4 h.

The most common indications for SC insulin as declared by the centres were previous treatment by SC insulin (18/64), patients eating by mouth (8/64), and relay after IV insulin administration (5/64). Only 3 (4.7%) ICUs indicated they prescribe first line SC insulin in mild hyperglycaemias. Only one centre declared adapting blood glucose target to the patient’s HbA1c value. Five centres (7.8%) reported targeting different blood glucose ranges in patients with and without diabetes. Among the 64 centres for which these data were available, 40 (62.5%) declared having a protocol for SC insulin administration.

### Patients characteristics

Among 906 eligible patients, 13 (1.4%) were excluded because of incomplete data. Therefore, we analysed 893 patients (Additional file 1: Fig. S4).

Patients’ characteristics are presented in Table 1. The mean age (SD) of the study population was 61.6 [14] yrs.

**Table 1:** Patients’ characteristics

	Overall (*n* = 893)	Hospitalization < 7 days (*n* = 517)	Hospitalization ≥ 7 days (*n* = 376)	*P* value ^a^	Missing (%)^b^
**Demographics**					
Age, *mean (SD)*	61.6 (14.0)	61.9 (14.8)	61.3 (12.9)	0.51	0
Male	597 (66.9)	336 (65.0)	261 (69.4)	0.19	0
Days since admission	6 [2, 15]	3 [1, 5]	18 [12, 28]	< 0.001	0
Body mass index (kg/m^2^), *mean (SD)*	28.4 (8.0)	28.3 (7.6)	28.7 (8.5)	0.47	4.0
Body weight (kg), *mean (SD)*	81.6 (22.7)	81.0 (22.0)	82.3 (23.8)	0.41	1.1
SAPS2, *mean (SD)*	45.9 (19.3)	44.6 (19.3)	47.7 (19.2)	0.017	1.9
**Reason for admission**					
Septic shock	94 (10.5)	55 (10.6)	39 (10.4)	0.99	0
Other shock	53 (5.9)	22 (4.3)	31 (8.2)	0.019	0
Acute respiratory failure	485 (54.3)	265 (51.3)	220 (58.5)	0.037	0
Coma	80 (9.0)	45 (8.7)	35 (9.3)	0.85	0
Stroke	24 (2.7)	16 (3.1)	8 (2.1)	0.50	0
Cardiac arrest	61 (6.8)	41 (7.9)	20 (5.3)	0.16	0
Head trauma	8 (0.9)	1 (0.2)	7 (1.9)	0.024	0
Other trauma	10 (1.1)	4 (0.8)	6 (1.6)	0.41	0
Acute liver failure	7 (0.8)	3 (0.6)	4 (1.1)	0.67	0
Acute kidney failure	47 (5.3)	36 (7.0)	11 (2.9)	0.012	0
Surgery	60 (6.7)	42 (8.1)	18 (4.8)	0.12	0
Other	81 (9.1)	46 (8.9)	35 (9.3)	0.93	0
**Chronic illnesses**					
Chronic arterial hypertension	425 (47.6)	239 (46.2)	186 (49.5)	0.31	0
Chronic cardiac insufficiency	116 (13.0)	69 (13.3)	47 (12.5)	0.76	0
Chronic lung insufficiency	174 (19.5)	105 (20.3)	69 (18.4)	0.55	0
Chronic kidney disease	105 (11.8)	67 (13.0)	38 (10.1)	0.21	0
Hemodialysis	26 (2.9)	20 (3.9)	6 (1.6)	0.07	0
Cancer or hematologic malignancy	116 (13.0)	75 (14.5)	41 (10.9)	0.13	0
Solid organ transplant	29 (3.2)	12 (2.3)	17 (4.5)	0.08	0
Type 1 diabetes	8 (0.9)	5 (1.0)	3 (0.8)	> 0.99	0
Type 2 diabetes	204 (22.8)	104 (20.1)	100 (26.6)	0.024	0
Cirrhosis	24 (2.7)	11 (2.1)	13 (3.5)	0.29	0
**Chronic medications**					
Long-acting insulin	55 (6.2)	23 (4.4)	32 (8.5)	0.019	0
Short-acting insulin	49 (5.5)	19 (3.7)	30 (8.0)	0.008	0
Intermediate insulin	4 (0.4)	2 (0.4)	2 (0.5)	> 0.99	0
**Characteristics on study day**					
Temperature (°C)	37.0 [36.4, 37.5]	36.8 [36.3, 37.4]	37.1 [36.6, 37.6]	< 0.001	2.2
Infection	556 (62.3)	299 (57.8)	257 (68.4)	0.003	0
Antibiotic	484 (54.2)	255 (49.3)	229 (60.9)	< 0.001	0
Vasopressor	240 (26.9)	145 (28.1)	95 (25.2)	0.44	0
Mechanical ventilation	583 (67.5)	289 (55.9)	294 (78.2)	< 0.001	0
Enteral nutrition	429 (48.0)	155 (30.0)	274 (72.9)	< 0.001	0
Oral nutrition	231 (25.9)	176 (34)	55 (14.6)	< 0.001	0
Parenteral nutrition	91 (10.2)	38 (7.4)	53 (14.1)	0.001	0
2.5% Glucose administration	60 (6.7)	26 (5.0)	34 [9]	0.021	0
5% Glucose administration	580 (64.9)	332 (64.2)	248 (66)	0.422	0
10% Glucose administration	40 (4.5)	34 (6.6)	6 (1.6)	< 0.001	0
AKI KDIGO > 0	257 (28.8)	144 (27.9)	113 [30]	0.50	0
Corticosteroids within 3 days	355 (39.8)	224 (43.3)	131 (34.8)	0.015	0
Glucagon	2 (0.2)	1 (0.2)	1 (0.3)	> 0.99	0
SOFA score on study day	5 [3, 8]	5 [2, 8]	5 [3, 8.25]	0.11	0.1

Results expressed as number (percentage) or median (interquartile range) unless otherwise specified

*AKI* acute kidney injury; *KDIGO* Kidney disease improving global outcomes [31]; *IQR* Interquartile range; *SAPS2* Simplified acute physiology score [32]; *SOFA* sepsis related organ failure [18]

^a^Between-group differences in proportions were tested by Fisher exact test, excluding patients with missing values

^b^Percentage of patients with missing value (over 893 patients)

The majority of patients (732/893 [82.0%]) were monitored over a full 24-h period. The remaining patients, who were admitted (80 [8.9%]), discharged (80 [8.9%]), or both (1 [0.1%]) on the calendar day of the study, were also included in the analyses.

### Administration of insulin

Overall, 408/893 (45.7%) patients received insulin. Among them, 178/408 (43.6%) were diabetic. In counterpart, 35/215 (16.3%) of diabetic patients had no insulin.

Intravenous (IV) insulin was the most used form of insulin regardless of the period and was prescribed alone to 255/408 (62.5%) patients, whereas the SC route alone was used in 126/408 (30.9%) patients, and both IV and SC routes were used in 27/408 (6.6%) patients (Table 2).

**Table 2:** Insulin management

	Overall (n = 408)	Hospitalization < 7 days (*n* = 199)	Hospitalization ≥ 7 days (*n* = 209)	*P* value
**IV insulin**	282 (69.1)	136 (68.3)	146 (69.9)	0.82
Total amount of IV insulin (IU/day) in patient receiving IV insulin (median (IQR))	49 [24, 83]	40 [21, 83]	56 [30, 82]	0.027
**SC insulin**	153 (37.5)	79 (39.7)	74 (35.4)	0.43
Short-acting insulin	133 (32.6)	74 (37.2)	59 (28.2)	0.07
Total amount of short-acting insulin (IU/day) in patient receiving SC insulin (median (IQR))	12 [8, 25]	14 [8, 22]	12 [8, 28]	0.89
Long-acting insulin	52 (12.7)	15 (7.5)	37 (17.7)	0.003
Total amount of long-acting insulin (IU/day) in patient receiving SC insulin (median (IQR))	30 [16, 45]	20 [12, 30]	30 [18, 68]	0.017
Intermediate insulin	4 (1.0)	2 (1.0)	2 (1.0)	> 0.99
Total amount of intermediate insulin (IU/day) in patient receiving SC insulin (median (IQR))	37 [32, 49]	37 [36, 39]	50 [37, 63]	> 0.99
Patients who had previous treatment with IV insulin before SC insulin administration	60 (39.2)	21 (26.6)	39 (52.7)	0.002
**IV + SC insulin during the study day**	27 (6.6)	16 (8.0)	11 (5.3)	0.35
**SC long-acting + short-acting during the study day**	35 (8.6)	11 (5.5)	24(11.5)	0.035

Results expressed as number (percentage) or median (interquartile range)

### Factors associated with the blood glucose level

Linear mixed models dichotomizing the study population according to whether patients were monitored on day 1 vs after day 1, or before or on day 3 vs after day 3, both had significantly higher deviance (*P* < 0.0001) than the model using < day 7 vs  ≥ day 7, as assessed by the likelihood ratio test. Therefore, we retained the latter model using < day 7 (Period 1) vs  ≥ day 7 (Period 2).

**Fig. 2:**
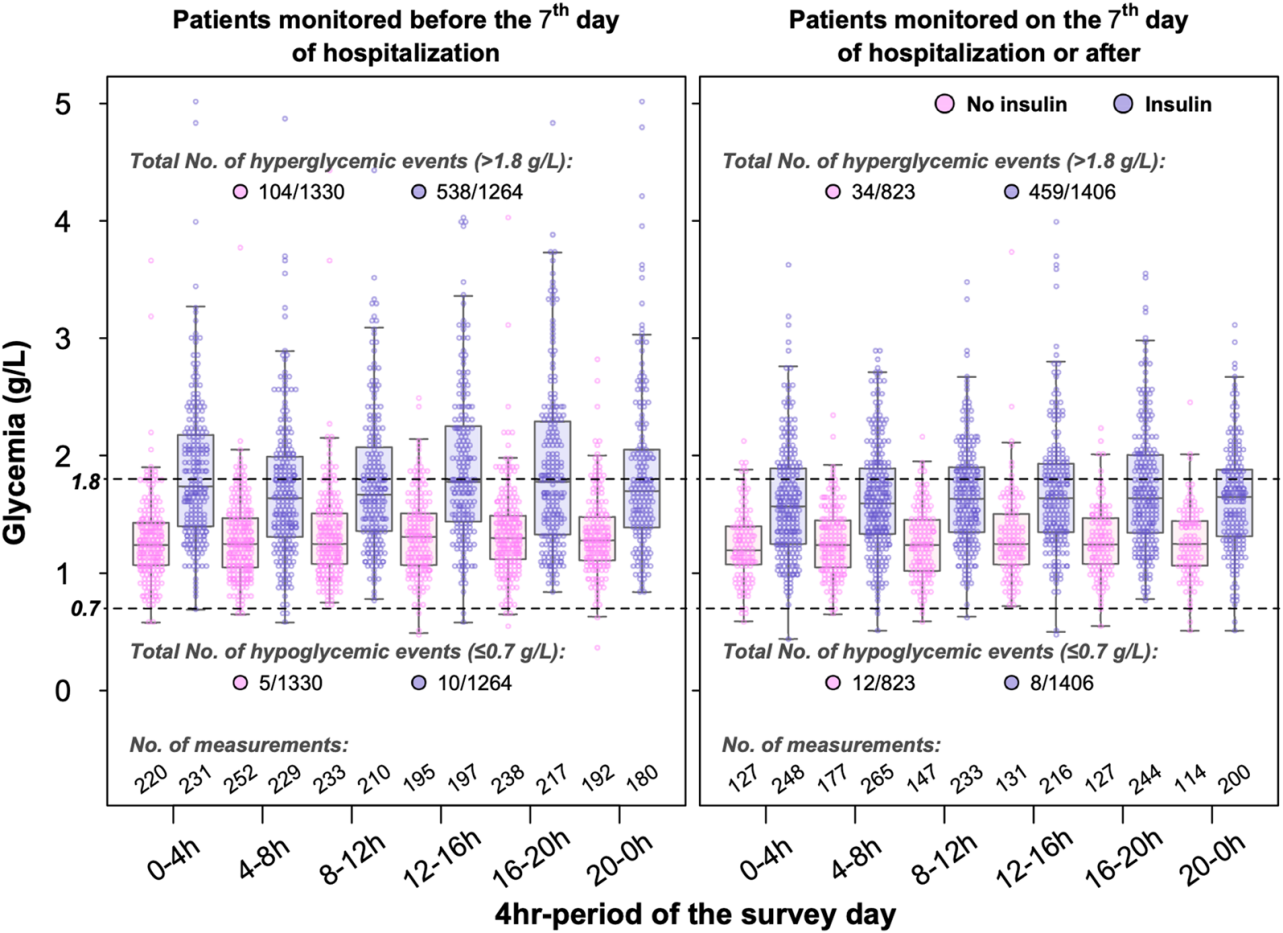
Glycaemia by 4-h periods in patients receiving insulin or not. All individual glycaemias appear as small pink or blue empty circles. Boxplots represent the median and interquartile range of glycaemia in each time slot

The individual mean glycemia over the study day did not differ between patients under insulin or no (1.57 g/L ± 0.62 vs 1.51 g/L ± 0.48; *P* > 0.99), but its variance was significantly higher in patients under insulin (*P* < 0.0001) (Additional file 1: Table S3). Densities of probability of mean individual glycaemia during Period 1 and 2 are represented in Additional file 1: Fig. S5. There was a strong interaction between the period of ICU stay (Period1 or Period2) and the use of insulin (*P* = 0.0004). This strong interaction led us to perform and report the remaining analyses for Period 1 and Period 2 separately (Fig. 2).

**Fig. 3:**
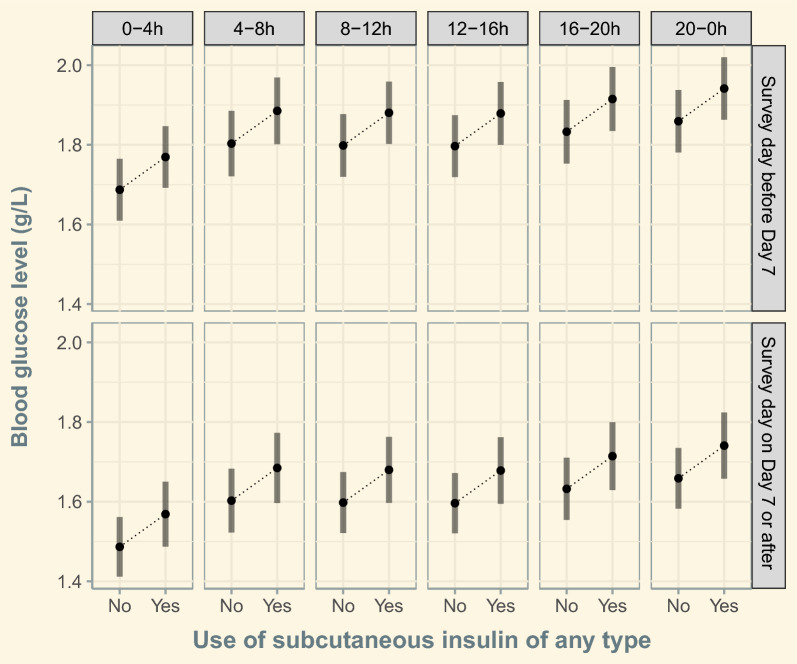
Estimated marginal mean of blood glucose level according to the time slots, the period of observation and the use of subcutaneous insulin of any type in patients receiving insulin. Black circles represent the estimated marginal mean of glycaemia. Vertical bars represent the 95% confidence interval of the mean

During Period 1, the variables “diabetes”, “use of IV insulin”, “use of SC insulin”, “SOFA score” and time slots “16-20 h” and “20-0 h”, were significantly associated with higher blood glucose level, and the variable “ongoing infection” was significantly associated with lower blood glucose levels (Additional file 1: Table S4). During Period 2 the variables “diabetes”, “use of IV insulin”, “use of SC insulin”, and “SOFA score” were significantly associated with higher blood glucose levels (Additional file 1: Table S5). In the population restricted to the 408 patients receiving insulin via any route, linear mixed regression analysis weighted by propensity score-based IPTW (see detailed method in Additional file 1), showed that the use of SC insulin of any type was associated with higher blood glucose levels (estimated marginal mean difference: 0.08 g/L [95% CI 0.02; 0.14], *P* = 0.004), a finding that was consistent across time slots and periods, as illustrated in Fig. 3. In this population, the use of SC long-acting insulin, which was more frequent in Period 2 than in Period 1 (37/209, 17.7% vs 15/199, 7.5%; *P* = 0.003), was not associated with higher mean blood glucose levels (estimated marginal mean difference: 0.06 g/L [95% CI −0.04; 0.15], *P* = 0.27).

### Glycaemic control

**Fig. 4:**
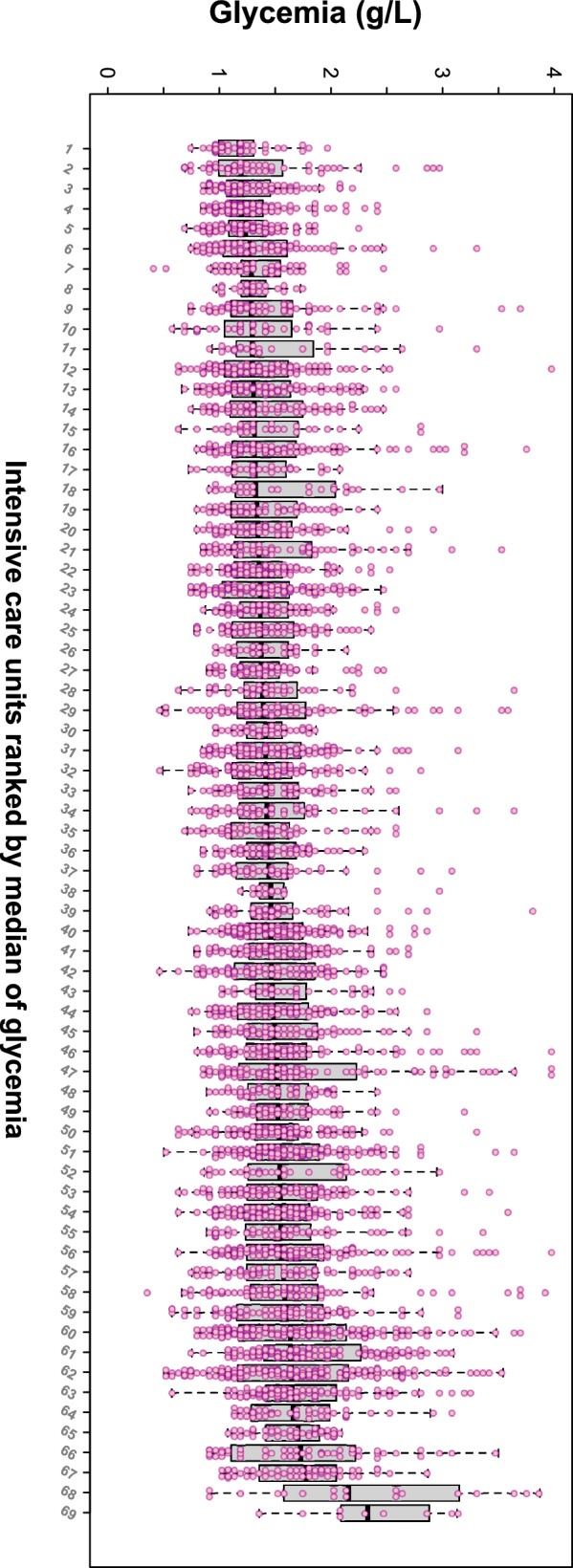
Distribution of glycaemia in each intensive care unit. Boxplots represent the median and interquartile range of glycaemia observed in each ICU are provided. Small blue points represent all individual glycaemias. The distribution of glycaemia varied significantly between intensive care units (*P* < 0.0001 by Kruskal–Wallis test), even when excluding the two ICUs with the highest median of glycaemia

Among 4900 glucose tests performed in 893 patients, 4823 glucose values were collected (Additional file 1: Table S3). The distribution of glycaemia varied significantly across ICUs (*p* < 0.0001) (Fig. 4). The interval between two blood glucose tests had a median of 3.4 h [IQR: 2.7, 4.0], and was significantly shorter in patients under IV insulin than in patients receiving SC insulin (3.4 h [3.0, 4.0] vs 4.0 h [4.0, 4.8]; *P* < 0.0001), while it did not differ significantly between Period 1 and Period 2 (3.4 h [IQR: 2.7, 4.0] for both; *P* = 0.94).

The number of blood glucose tests performed per patient was positively correlated with the SOFA score value on the study day (P < 0.0001) (Additional file 1: Fig. S6, S7).

### Association of insulin protocols with the blood glucose level

The proportion of blood glucose values above the target range set by protocol *among patients receiving IV insulin* for whom these data were available was 767/1681 (45.6%). This proportion was higher in Period 1 than in Period 2 (406/821 [49.5%] vs 345/839 [41.1%]; *P* = 0.0008).

In this population, introducing the high target value set per protocol in the linear mixed models did not better explain the blood glucose level during Period 1 (*P* = 0.17 by likelihood ratio test), but did so during Period 2 (*P* = 0.023). During this latter period, the increase in blood glucose value associated with each increment of 0.1 g/L in the high target value was 0.025 g/L, as illustrated in Additional file 1: Fig. S8.

### Individual glucose variability on the study day

The individual glucose variability could be calculated for only 831 (93.1%) patients, because 62 patients had zero or only one blood glucose value measured on the study day. The individual glucose variability was significantly higher in patients receiving insulin via any route (19.4 [IQR: 13.4–28.4] vs 11.4 [IQR: 7.6–17.1]; P < 0.0001). The whole results regarding glucose variability are presented in Additional file 1: Figs. S9–S12.

### Hypo- and hyperglycaemic events

As illustrated in Fig. 2, and shown in Additional file 1: Tables S3, 35 events of hypoglycaemia (≤ 0.7 g/L) were observed in 26 (2.9%) patients, including only one instance of glycaemia ≤ 0.4 g/L. The numbers of hypoglycaemias and hyperglycaemias per patient were positively correlated with the number of blood glucose tests performed per patient over the study day (see Additional file 1: Figs. S13 and S14).

There were 1135 hyperglycaemic events (> 1.8 g/L) observed in 402 (45.0%) patients. The proportion of blood glucose values > 1.8 g/L upon the number of tests performed was higher in Period1 (642 [24.7%]) than in Period2 (493 [22.1%]) (P = 0.032), a finding that was consistent across the patient subsets formed according to the known existence of diabetes and the use of insulin (Additional file 1: Table S6).

### Factors associated with the number of hyperglycaemias observed per patient

Insulin use, diabetes, and Period1 were associated with significantly increased counts of hyperglycaemic events (glycaemia > 1.8 g/L) as yielded by negative binomial regression (Additional file 1: Table S7).

In the subset of patients receiving insulin, the median number of hyperglycaemias in patients who were receiving SC insulin of any type was significantly higher than in patients who were not (2 [1; 4] vs 0 [0; 1]; median difference: 2 [95%CI 2; 2]; P < 0.0001). Negative binomial regression with propensity score-based IPTW showed that the use of SC insulin was associated with higher counts of hyperglycaemia (> 1.8 g/L) (IRR: 3.45 [95%CI 2.97; 4.00]; P < 0.0001) (Additional file 1: Table S8). Similarly, the use of SC long-acting insulin was associated with a higher median number of hyperglycaemias per patient (2.5 [1; 4] vs 2 [1; 3]; median difference: 2.5 [95%CI 2; 4]; P < 0.0001) and with higher counts of hyperglycaemia (IRR: 3.58 [2.84; 4.52]; P < 0.0001) (Additional file 1: Table S9).

## Discussion

This multicentre, 1-day cross-sectional study in 69 French ICUs showed that almost half of the patients received insulin, that SC insulin, despite being not recommended, was used in more than one-third of insulin-treated patients, and that hypoglycaemia was infrequent, whereas at least one episode of hyperglycaemia was observed in 45% of the patients.

As observed in a previous British survey [22], a vast majority of ICUs declared having a written protocol for glucose control and insulin management. Most of the ICUs had different blood glucose targets than that proposed by the guidelines, with out of the recommendations thresholds triggering insulin modifications: 19/64 (29.7%) had a protocol with a blood glucose value < 0.7 g/L to stop insulin injection and 40/64 (62.5%) a blood glucose value > 1.5 g/L to start insulin [6-8]. However, we observed high mean blood glucose levels, numerous hyperglycaemic events and a very low rate of hypoglycaemia, suggesting protocols may not have been strictly applied. A 2-week survey in 29 ICUs in Australia and New Zealand reported similar results, reporting higher glycaemias than protocols targets asserted by physicians [17]. A 1-day survey in 66 French ICUs documented similar results in 2013, with 58% of patients experiencing at least one hyperglycaemia event, but a higher occurrence of hypoglycaemia (15%) [23]. Our results showing a slightly improved glucose control than in 2013 may be related to practices enhancement and a better attention paid to glycaemia, but the discrepancies between targeted and observed glycaemias argue against this hypothesis.

We observed intervals between blood glucose measurements that were longer than recommended, even at the early phase, far from the guidelines suggestion to monitor glycaemia every 2 h. We can point out that the median number of 6 blood glucose tests per day did not differ from the previous French practice study by Orban et al. (6[4;9] blood glucose tests per day) but was higher than in the Krinsley et al. cohort (4.5 blood glucose tests per day in non-diabetic patients) [23, 24]. The correlation between hypo or hyperglycaemia events and higher blood glucose testing is interesting and reflects that it is less the delivery route than the glycaemic control which drives intervals between glycaemia measurements. This was in line with guidelines that recommend to assess glycaemia less or more frequently, depending on blood glucose stability [6].

Severe and even moderate hypoglycaemias were rare events, possibly because guidelines recommend to absolutely avoid low blood glucose levels known to be associated with increased mortality [5-8]. In line with this, a before–after study in 49 ICUs in Australia and New-Zealand showed a trend toward upper range of glycaemic control after the publication of the NICE–SUGAR trial, and less frequent hypoglycaemias [25]. One may assume that worldwide clinical practices evolved the same way, despite a meta-analyse showing no effect of different targeted blood glucose ranges on mortality [26]. However, the lower limit of blood glucose target remained lower than recommended in several ICUs in our study. In addition, one may assume a discrepancy between real-life glucose control and the strict application of protocols during trials, in which hypoglycaemia was more prevalent in control arms than that we reported in daily practice [5, 27].

As expected, higher glycaemias were associated with higher SOFA score, i.e., more stress hyperglycaemia and insulin dysregulation. Patients receiving insulin had higher glycaemias, strengthening the idea that insulin was certainly used with caution to reduce the risk of hypoglycaemia. Another explanation might be that stress hyperglycaemia is hard to control due to insulin resistance. However, the fact that tight glycaemic control in randomised trials actually achieved lower glycaemic ranges, and the persistent association between high blood glucose levels and insulin use in Period 2 in our study do not support that hypothesis.

Consistent with the high number of blood glucose values out of the targeted ranges, we observed a glucose variability > 15% in half of the patients. Interestingly, it did not differ between Period 1 and 2, which contrast with the association we reported between hyperglycaemic events and Period 1. The increased glucose variability in diabetic patients and those treated by insulin was expected and consistent with the significant association to a higher rate of hyperglycaemias in the linear mixed models. It may support the fact that blood glucose target could be hard to reach due to insulin resistance in critically ill patients. Glucose variability did not differ significantly whether the patients received SC or IV insulin, while long-acting SC insulin was associated with more hyperglycaemias. These discrepancies should call for caution in interpreting blood glucose variability, given the 1-day design of the study, and instead prompt a focus on the crude percentage of hypoglyceamic and hyperglycaemic events. 

Most published ICU studies provided little data about insulin administration, probably assuming the IV route was mandatory. However, SC delivery was not uncommon in our survey. The high rate of SC insulin use contrasts with reported reasons to begin SC insulin. Whereas only 3 ICUs indicated they allow first-line SC insulin, 39.2% of the patients under SC insulin never had IV insulin before. The use of SC instead of IV insulin, including during early stage of ICU stay, may have several explanations, such as less blood glucose measurements, reduced workload, and fear of hypoglycaemia or insulin dosing errors with continuous IV infusion [28]. These explanations are only hypothesis-based, as the reasons for SC insulin use could not be recorded for each patient. In a pilot study of SC vs IV insulin in 58 non-diabetic trauma patients, time to achieve blood glucose target (goal was tight control) was faster with IV than SC insulin [15], and glycaemias were lower with IV insulin, like in our study. A retrospective study reported that patients treated with SC insulin had more hyperglycaemias (52.2% vs 35.8%) but also more frequent hypoglycaemias (2.1% vs 1.2%) than those treated with IV insulin. However, the observations from this two-ICU, single-hospital study are hard to generalise, because most patients under SC insulin were predominantly hospitalised in the medical ICU and those treated with IV insulin hospitalised in the surgical ICU. In a recent randomized controlled pilot study the addition of a fixed 15 IU/day dose of long-acting insulin to IV infusion led to more and longer hypoglycaemias than in the control group [13]. In our study, long-acting insulin, despite being prescribed at higher doses, was associated with more hyperglycaemias but similar mean blood glucose compared with patients under other types of insulin. End dose glycaemic rebound might be an explanation. In a small-size randomized study, stable post-operative critical care patients receiving parenteral nutrition had similar blood glucose levels and hyperglycaemia rates whether they underwent short or long-acting insulin therapy. Hyperglycaemia were less frequent (11%) than in our 1-day study, and there was no association with long-acting insulin use [29].

### Implications for clinical practice

Although it is only a hypothesis, the frequent exposition of critically ill patients to hyperglycaemia in our study may be seen as an effect of the "fear of hypoglycaemia” following the NICE–SUGAR trial [5]. Previous reports corroborated this hypothesis, as even when the tight glycaemic control was assumed to reduce mortality, its implementation in ICUs was not the rule [17, 22]. The gap between targeted glycaemias, ruled by protocols with a lower limit often below what guidelines suggested, and glycaemias actually reported in our study should question the importance physicians gave to blood glucose control. Yet, hyperglycaemia, like hypoglycaemia, is associated with higher mortality in specific patients, and should not be neglected [2]. The potential difficulty to regulate blood glucose with SC insulin in critically ill patients raises safety concerns about its frequent use we reported and the associated hyperglycaemic events. Our survey highlights the need for clinicians to pay attention to blood glucose management in their daily practice and question the way to achieve it, the place of SC insulin and to what extent protocols are correctly applied.

### Perspectives

Randomized trials are mandatory to assess whether SC insulin should be abandoned or not to the benefit of IV insulin exclusively. The population that might have glycaemia in targeted range with SC insulin remains to be defined. Further studies, like the ongoing dedicated French survey conducted by the FICS (ID-RCB: 2022-A01304-39), should also focus on nurse burden of work regarding blood glucose management and insulin delivery route.

### Limitations

Due to its observational design, there is an inherent risk of residual confounding in our study, despite the adjustments made. Details regarding regular or shorter-acting insulin were not collected, but the consequences are limited as hypoglycaemic events were infrequent [30]. We do not know in which proportions patients under SC insulin had a sliding scale protocol, basal-bolus or prandial insulin [7]. The way blood glucose samples were obtained (capillary or arterial blood samples) was not recorded.

## Conclusions

Practices regarding blood glucose management vary widely among French ICUs. Administration of short or long-acting SC insulin is not unusual, and its role deserves to be better defined. The use of protocolized insulin algorithms failed to prevent hyperglycaemic events.

## Supplementary Information

**Additional file 1: Fig. S1 **Geographical location of the participating intensive care units. **Table S1.** Repartition of the patients included according to the ICU. **Table S2.** Characteristics of the ICU. **Fig. S2.** Thresholds to start, resume or stop insulin. **Fig. S3.** Standard and lowest intervals between blood glucose tests. **Fig. S4.** Flow chart. **Table S3. **Glycemic control. **Fig. S5.** density of probability of mean individual glycaemia. **Table S4.** Linear mixed model—Period 1. **Table S5.** Linear mixed model—Period 2. **Fig. S6.** Number of blood glucose tests performed on the study day during Period 1 according to the SOFA score. **Fig. S7**. Number of blood glucose tests performed on the study day during Period 2 according to the SOFA score. **Fig. S8.** blood glucose value of patient under IV insulin during Period 2 according to the upper limit of the ICU’s blood glucose management protocol. **Fig. S9.** Individual glucose variability according to the periodand types of insulin used, in all patients. **Fig. S10.** Individual glucose variability according to the periodand types of insulin used, in patients without diabetes. **Fig. S11.** Individual glucose variability according to the periodand types of insulin used, in patients with diabetes. **Fig. S12**. Repartition of the individual glucose variability according to the period, existence of diabetes, and use of subcutaneous insulin, in patients receiving insulin of any type. **Fig. S13**. Number of blood glucose tests performed according to the number of hypoglycaemias. **Fig. S14.** Number of blood glucose tests performed according to the number of hyperglycaemias. **Table S6.** Hyperglycemic events. **Table S7.** Incidence rate ratio of hyperglycemia. **Table S8.** Incidence rate ratio of hyperglycemia. **Table S9.** Incidence rate ratio of hyperglycemia

## Data Availability

The data sets used and/or analysed during the current study are available from the corresponding author on reasonable request.

## Abbreviations

AKI: Acute kidney injury
CRF: Case report form
ICU: Intensive care unit
IPTW: Propensity score-based inverse probability weighting
IQR: Interquartile range
IRR: Incidence rate ratio
IV: Intravenous
KDIGO: Kidney disease improving global outcomes
OR: Odds-ratio
SAPS2: Simplified acute physiology score
SC: Subcutaneous
SD: Standard deviation
SFAR: French Society of Anaesthesia and Intensive Care
SOFA: Sequential organ failure assessment
SRLF: French Intensive Care Society
