# Supplementary material for: Insulin therapy and blood glucose management in critically ill patients: a 1-day cross-sectional observational study in 69 French intensive care units
Source: Ann Intensive Care. 2023 Jun 17;13:53. doi: 10.1186/s13613-023-01142-9 (PMC10276797; doi:10.1186/s13613-023-01142-9)
Supplement: Supplementary file 1 — Additional file 1: Fig. S1 Geographical location of the participating intensive care units. Table S1. Repartition of the patients included according to the ICU. Table S2. Characteristics of the ICU. Fig. S2. Thresholds to start, resume or stop insulin. Fig. S3. Standard and lowest intervals between blood glucose tests. Fig. S4. Flow chart. Table S3. Glycemic control. Fig. S5. density of probability of mean individual glycaemia. Table S4. Linear mixed model—Period 1. Table S5. Linear mixed model—Period 2. Fig. S6. Number of blood glucose tests performed on the study day during Period 1 according to the SOFA score. Fig. S7. Number of blood glucose tests performed on the study day during Period 2 according to the SOFA score. Fig. S8. blood glucose value of patient under IV insulin during Period 2 according to the upper limit of the ICU’s blood glucose management protocol. Fig. S9. Individual glucose variability according to the periodand types of insulin used, in all patients. Fig. S10. Individual glucose variability according to the periodand types of insulin used, in patients without diabetes. Fig. S11. Individual glucose variability according to the periodand types of insulin used, in patients with diabetes. Fig. S12. Repartition of the individual glucose variability according to the period, existence of diabetes, and use of subcutaneous insulin, in patients receiving insulin of any type. Fig. S13. Number of blood glucose tests performed according to the number of hypoglycaemias. Fig. S14. Number of blood glucose tests performed according to the number of hyperglycaemias. Table S6. Hyperglycemic events. Table S7. Incidence rate ratio of hyperglycemia. Table S8. Incidence rate ratio of hyperglycemia. Table S9. Incidence rate ratio of hyperglycemia [file 13613_2023_1142_MOESM1_ESM.docx]

**Additional File**

**Title**:

Insulin therapy and blood glucose management in critically ill patients: a one-day cross-sectional observational study in 69 French intensive care units.

**Authors**:

Maxime Desgrouas, Julien Demiselle, Laure Stiel, Vincent Brunot, Rémy Marnai, Sacha Sarfati, Maud Fiancette, Fabien Lambiotte, Arnaud W. Thille, Maxime Leloup, Sébastien Clerc, Pascal Beuret, Anne-Astrid Bourion, Johan Daum, Rémi Malhomme, Ramin Ravan, Bertrand Sauneuf, Jean-Philippe Rigaud, Pierre François Dequin, Thierry Boulain

**Corresponding author**:

Maxime Desgrouas

Médecine Intensive Réanimation

Centre Hospitalier Régional d’Orléans

14 avenue de l’hôpital

45100 Orléans

+33 (0)2 38 51 44 46

[maxime.desgrouas@chr-orleans.fr](mailto:maxime.desgrouas@chr-orleans.fr)

**Methods for propensity score-based inverse probability of treatment weighting to assess the association of subcutaneous insulin use with the blood glucose level.**

First, we built 50 imputed datasets of the study population by using multivariable imputation by chained equations [1], to replace missing values (see Table 1 of the main manuscript) of body weight, BMI, body temperature, SAPS2 score, and SOFA score. We used the predictive mean matching method to impute those continuous variables, using the *mice* package of R. All patients’ baseline characteristics were used as predictors.

Second, for each patient we calculated two distinct propensity scores (PS) [2], i.e., the probability of being prescribed subcutaneous (SC) insulin of any type and the probability of being prescribed SC long-acting insulin, by using multivariable logistic regression and the following independent baseline covariables: Type of ICU, age, sex, SAPS2 score on admission, cause of admission, underlying comorbidities, previous regular treatment by SC insulin, ongoing infection on study day, use of vasopressors, mechanical ventilation or not, artificial nutrition via enteral or intravenous (IV) route, oral alimentation, number of days since ICU admission, SOFA score on study day.

Each PS (one for the propensity to receive SC insulin of any type, one for the propensity to receive SC long-acting insulin) for a given patient was defined as its mean over the 50 imputed datasets.

Then, in the subset of patients receiving insulin via either route (IV or SC), we use each PS to weight the analyses (linear mixed models, see Methods in the main manuscript) performed to assess the association of the blood glucose level with the use of SC insulin of any type, and with the use of SC long-acting insulin, through stabilized inverse probability of treatment weighting (IPTW) [3].

PS-based IPTW was also used for examining the association of the use of SC insulin with the number of hyperglycemic events per patient through multivariable mixed-effect negative binomial regression analysis, in the subset of patients receiving insulin.

**Fig. S1 Geographical location of the participating intensive care units**

**
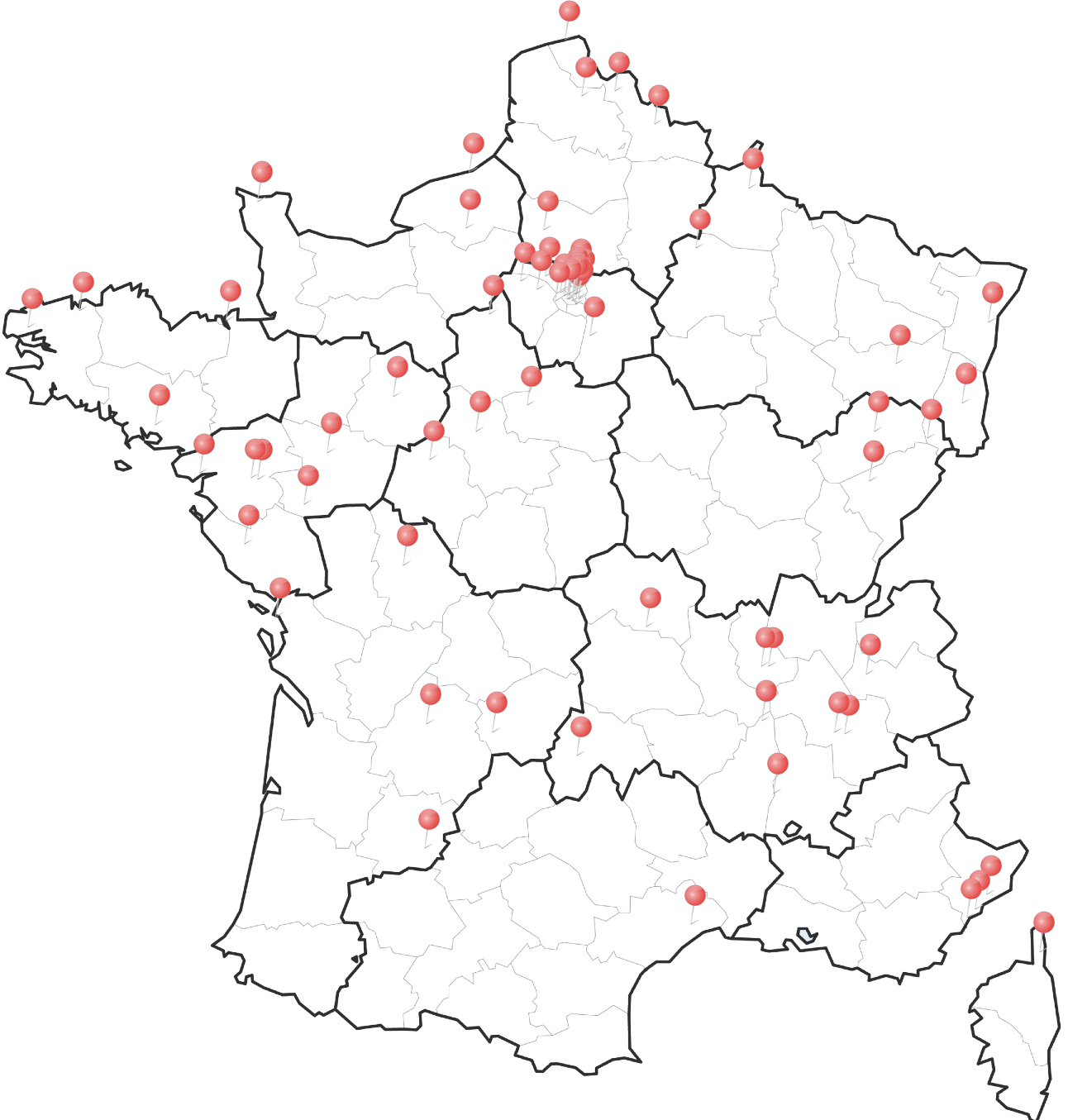
**

Fig. S1 caption: each red needle represents the location of one intensive care unit.

**Table S1 – Repartition of the patients included according to the ICU.**

|  | **Overall** | **Hospitalisation < 7 days** | **Hospitalisation ≥ 7 days** |
| --- | --- | --- | --- |
| **Number of patients** | 893 | 517 | 376 |
| **Centre** (%) |  |  |  |
| Agen | 11 (1.2) | 3 (0.6) | 8 (2.1) |
| Angers | 28 (3.1) | 14 (2.7) | 14 (3.7) |
| Annonay | 6 (0.7) | 5 (1.0) | 1 (0.3) |
| Antibes | 8 (0.9) | 7 (1.4) | 1 (0.3) |
| Argenteuil | 9 (1.0) | 2 (0.4) | 7 (1.9) |
| Aulnay | 8 (0.9) | 4 (0.8) | 4 (1.1) |
| Aurillac | 8 (0.9) | 5 (1.0) | 3 (0.8) |
| Bastia | 12 (1.3) | 5 (1.0) | 7 (1.9) |
| Beauvais | 9 (1.0) | 4 (0.8) | 5 (1.3) |
| Besancon | 13 (1.5) | 9 (1.7) | 4 (1.1) |
| Bethune | 10 (1.1) | 6 (1.2) | 4 (1.1) |
| Blois | 2 (0.2) | 2 (0.4) | 0 (0.0) |
| Brest | 15 (1.7) | 13 (2.5) | 2 (0.5) |
| Chambery | 16 (1.8) | 13 (2.5) | 3 (0.8) |
| Charleville_Mezieres | 9 (1.0) | 5 (1.0) | 4 (1.1) |
| Cherbourg | 6 (0.7) | 2 (0.4) | 4 (1.1) |
| Cholet | 9 (1.0) | 4 (0.8) | 5 (1.3) |
| Cochin | 23 (2.6) | 16 (3.1) | 7 (1.9) |
| Diaconesses_Croix_St_Simon | 10 (1.1) | 8 (1.5) | 2 (0.5) |
| Dieppe | 6 (0.7) | 5 (1.0) | 1 (0.3) |
| Dreux | 11 (1.2) | 3 (0.6) | 8 (2.1) |
| Dunkerque | 12 (1.3) | 10 (1.9) | 2 (0.5) |
| Epinal | 7 (0.8) | 5 (1.0) | 2 (0.5) |
| Foch | 17 (1.9) | 8 (1.5) | 9 (2.4) |
| Garches | 12 (1.3) | 5 (1.0) | 7 (1.9) |
| Gonesse | 15 (1.7) | 9 (1.7) | 6 (1.6) |
| Grenoble cardiac ICU | 10 (1.1) | 8 (1.5) | 2 (0.5) |
| Grenoble medical ICU | 7 (0.8) | 3 (0.6) | 4 (1.1) |
| Gustave_Roussy | 7 (0.8) | 7 (1.4) | 0 (0.0) |
| La_Roche_sur_Yon | 17 (1.9) | 11 (2.1) | 6 (1.6) |
| La_Rochelle | 15 (1.7) | 8 (1.5) | 7 (1.9) |
| Le_Mans | 19 (2.1) | 11 (2.1) | 8 (2.1) |
| Lomme | 11 (1.2) | 10 (1.9) | 1 (0.3) |
| Louis_Mourier | 7 (0.8) | 5 (1.0) | 2 (0.5) |
| Lyon cardiac ICU | 13 (1.5) | 8 (1.5) | 5 (1.3) |
| Lyon medical ICU | 16 (1.8) | 12 (2.3) | 4 (1.1) |
| Mantes_la_Jolie | 4 (0.4) | 0 (0.0) | 4 (1.1) |
| Melun | 14 (1.6) | 7 (1.4) | 7 (1.9) |
| Mondor | 18 (2.0) | 8 (1.5) | 10 (2.7) |
| Montelimar | 11 (1.2) | 6 (1.2) | 5 (1.3) |
| Montpellier | 20 (2.2) | 9 (1.7) | 11 (2.9) |
| Morlaix | 8 (0.9) | 4 (0.8) | 4 (1.1) |
| Mulhouse | 21 (2.4) | 13 (2.5) | 8 (2.1) |
| Nantes medical ICU | 30 (3.4) | 21 (4.1) | 9 (2.4) |
| Nantes surgical ICU | 18 (2.0) | 12 (2.3) | 6 (1.6) |
| Nice | 12 (1.3) | 7 (1.4) | 5 (1.3) |
| Orleans | 26 (2.9) | 14 (2.7) | 12 (3.2) |
| Paris_Saint_Joseph | 14 (1.6) | 9 (1.7) | 5 (1.3) |
| Perigueux | 14 (1.6) | 9 (1.7) | 5 (1.3) |
| Pitie_Salpetriere | 14 (1.6) | 8 (1.5) | 6 (1.6) |
| Pitie_Salpetriere_2 | 17 (1.9) | 5 (1.0) | 12 (3.2) |
| Poissy | 13 (1.5) | 9 (1.7) | 4 (1.1) |
| Poitiers | 16 (1.8) | 8 (1.5) | 8 (2.1) |
| Pontoise | 20 (2.2) | 4 (0.8) | 16 (4.3) |
| Reims | 13 (1.5) | 5 (1.0) | 8 (2.1) |
| Roanne | 11 (1.2) | 7 (1.4) | 4 (1.1) |
| Rouen | 19 (2.1) | 15 (2.9) | 4 (1.1) |
| Saint_Anne | 5 (0.6) | 2 (0.4) | 3 (0.8) |
| Saint_Antoine | 11 (1.2) | 9 (1.7) | 2 (0.5) |
| Saint_Louis | 10 (1.1) | 7 (1.4) | 3 (0.8) |
| Saint_Malo | 8 (0.9) | 4 (0.8) | 4 (1.1) |
| Saint_Nazaire | 12 (1.3) | 8 (1.5) | 4 (1.1) |
| Strasbourg | 26 (2.9) | 14 (2.7) | 12 (3.2) |
| Tours | 24 (2.7) | 8 (1.5) | 16 (4.3) |
| Trevenans | 3 (0.3) | 2 (0.4) | 1 (0.3) |
| Valenciennes | 17 (1.9) | 9 (1.7) | 8 (2.1) |
| Vannes | 9 (1.0) | 5 (1.0) | 4 (1.1) |
| Vesoul | 13 (1.5) | 8 (1.5) | 5 (1.3) |
| Vichy | 8 (0.9) | 6 (1.2) | 2 (0.5) |

**Table S2 – Characteristics of the ICU**

|  |  |  |  |  |  |
| --- | --- | --- | --- | --- | --- |
| **ICU type** (%) | N=69 |  |  |  |  |
| Cardiac | 2 (2.9) |  |  |  |  |
| Medical | 23 (33.3) |  |  |  |  |
| Surgical | 2 (2.9) |  |  |  |  |
| Neurological | 1 (1.4) |  |  |  |  |
| Mixed | 39 (56.5) |  |  |  |  |
| **Number of beds**, median [IQR] | 17 [12 ; 20] |  |  |  |  |
|  | **Overall** | **Hospitalisation < 7 days** | **Hospitalisation ≥ 7 days** | **P-value** | **Missing (%)** |
| **Number of patients** | 893 | 517 | 376 |  |  |
| **Number of patients by ICU type** (%) |  |  |  | 0.51 | 0.0 |
| Cardiac | 23 (2.6) | 16 (3.1) | 7 (1.9) |  |  |
| Medical | 396 (44.3) | 226 (43.7) | 170 (45.2) |  |  |
| Surgical | 31 (3.5) | 21 (4.1) | 10 (2.7) |  |  |
| Neurological | 5 (0.6) | 2 (0.4) | 3 (0.8) |  |  |
| Mixed | 438 (49.0) | 252 (48.7) | 186 (49.5) |  |  |

**Fig. S2: Thresholds to start, resume or stop insulin**

Fig. S2 caption: each purple diamond represents the threshold blood glucose value in g/L of one intensive care unit. Boxplots represent the median and interquartile range of threshold.

**Fig. S3: Standard and lowest intervals between blood glucose tests**

Fig. S3 caption: each purple diamond represents the interval in hours between two blood glucose tests in each intensive care unit. Boxplots represent the median and interquartile range of these intervals.

**Fig. S4: Flow chart
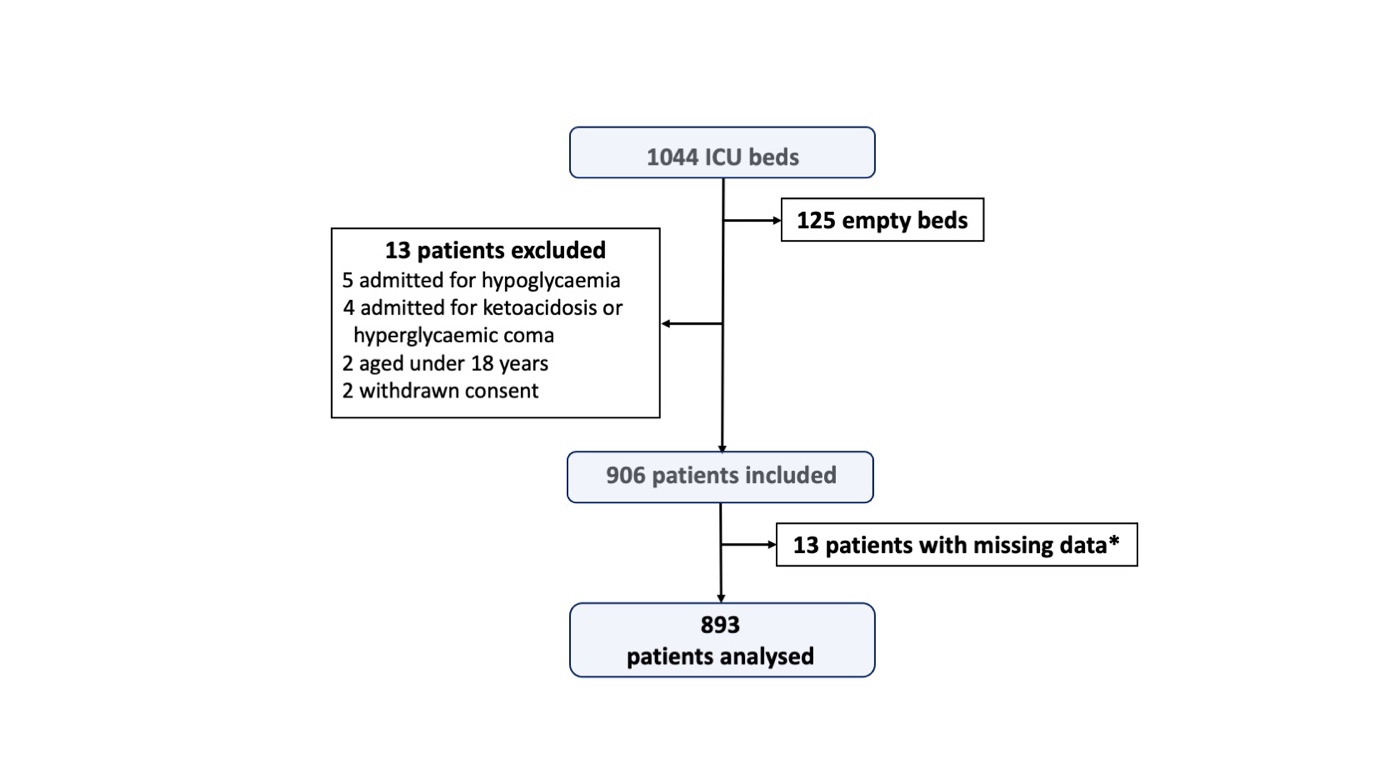
**

Fig. S4 caption: among the 1044 ICU beds available, 125 were empty, 13 patients had exclusion criteria and 13 patients missing data (*day of admission not recorded). Eventually, 893 patients were analysed.

**Table S3. Glycemic control.**

|  | **All**  **(n=893)** | **Insulin (n=408)** | **No Insulin (n=485)** |
| --- | --- | --- | --- |
| Total No. of blood glucose tests performed | 4900 | 2716 | 2184 |
| Total No. of blood glucose values reported | 4823 | 2670 | 2153 |
| No. of blood glucose test performed per patient (median (IQR)) | 6 (4; 7) | 6 (6; 8) | 5 (3; 6) |
| Patients with no blood glucose test performed | 15 (1.7%) | 0 (0.0%) | 15 (3.1%) |
|  |  |  |  |
| Mean individual glycemia over the study day, *in g/L* (mean (SD)) | 1.51 (0.43) | 1.57 (0.62) | 1.51 (0.48) |
| Individual glucose variability, *in %* (median (IQR)) | 14.8 (9.5; 22.9) | 19.4 (13.4; 28.4) | 11.4 (7.6; 17.1) |
|  |  |  |  |
| Number of hypoglycemias ≤0.7 g/L | 35  (35/4823=0.7%) | 13  (13/2670=0.5%) | 22  (22/2153=1.0%) |
| Number of hypoglycemias ≤0.4 g/L | 1 | 0 | 1 |
| Patients with at least 1 episode of hypoglycemia ≤ 0.7 d/L | 26 (2.9) | 12 (2.9) | 14 (2.9) |
| Including patients with hypoglycemia ≤0.4 g/L | 1 (0.1) | 0 (0) | 1 (0.2) |
| Patients with at least 1 episode of hypoglycemia treated with 30% glucose IV infusion | 21 (2.4) | 10 (2.5) | 11 (2.3) |
|  |  |  |  |
| Patients with at least 1 episode of hyperglycemia >1.8 g/L | 402 (45.0) | 315 (77.2) | 87 (17.9) |
| Number of hyperglycemias >1.8 g/L | 1135  (1135/4823=23.5%) | 997  (997/2670=37.3%) | 138  (138/2153=6.4%) |
| Patients with all glycaemias between 0.8 and 1.8 g/L | 447 (50.1) | 87 (21.3) | 360 (74.2) |
| Number of glycemias between [0.8 and 1.8 g/L] | 3590  (3590/4823=74.4%) | 1647  (1647/2670=61.7%) | 1943  (1943/2153=90.2%) |

Numbers within brackets are percentage (%), unless otherwise specified.

**Fig.S5: density of probability of mean individual glycaemia**

Fig. S5 caption: density of probability of mean individual glycaemia is represented in red during period 1, in blue during period 2. Value at the peak of density is the mode for each period.

**Table S4. Linear mixed model – Period 1.**

|  | **Mean difference in blood glucose level**  **(95% confidence interval), g/L** | **P-value** |
| --- | --- | --- |
| Diabetes (minus no diabetes) | 0.17 (0.12 ; 0.23) | <0.0001 |
| IV insulin use (minus no use of IV insulin) | 0.35 (0.30 ; 0.41) | <0.0001 |
| SC insulin use (minus no use of SC insulin) | 0.41 (0.34 ; 0.47) | <0.0001 |
| SOFA score (per 1-point increase) | 0.01 (0.006 ; 0.018) | <0.0001 |
| 16-20hr time slot (compared to the rest of the day) | 0.07 (0.007 ; 0.124) | 0.029 |
| 20-0hr time slot (compared to the rest of the day) | 0.08 (0.02 ; 0.13) | 0.006 |
| Infection | -0.05 (-0.102 ; -0.005) | 0.030 |

**Table S5. Linear mixed model – Period 2.**

|  | **Mean difference in blood glucose level**  **(95% confidence interval), g/L** | **P-value** |
| --- | --- | --- |
| Diabetes (minus no diabetes) | 0.16 (0.11; 0.21) | <0.0001 |
| IV insulin use (minus no use of IV insulin) | 0.26 (0.22 ; 0.31) | <0.0001 |
| SC insulin use (minus no use of SC insulin) | 0.26 (0.20 ; 0.32) | <0.0001 |
| SOFA score (per 1-point increase) | 0.008 (0.003 ; 0.013) | 0.0009 |

**Fig.S6: Number of blood glucose tests performed on the study day during Period 1 according to the SOFA score**

Fig. S6 caption: Boxplots represent the median and interquartile range of the number of blood glucose tests performed on the study day according to the SOFA score on the study day, ordered from 0 to ≥ 9.

**Fig. S7: Number of blood glucose tests performed on the study day during Period 2 according to the SOFA score**

Fig. S7 caption: Boxplots represent the median and interquartile range of the number of blood glucose tests performed on the study day according to the SOFA score on the study day, ordered from 0 to ≥ 9.

**Fig. S8: blood glucose value of patient under IV insulin during Period 2 according to the upper limit of the ICU’s blood glucose management protocol**

Fig. S8 caption: Boxplots represent the median and interquartile range of the individual glycaemias of patients under IV insulin during Period 2. Each purple cross represents an individual glycaemia.

**Glucose Variability**

The individual glucose variability (14.8% [IQR: 9.5-22.9]) did not differ between ICUs (P=0.11) nor between Period1 and Period2 (15.2 [IQR: 9.4-23.2] and 14.5 [IQR: 9.5-22.3], respectively; P=0.57). It was significantly higher in patients with diabetes (19.8 [IQR: 12.8-30.0] vs 13.6 [IQR: 9.0-20.0]; P<0.0001).

The percentage of patients in each class of individual glucose variability (<15%, 15-30%, and >30%) were 51.1%, 35.1%, and 13.7%, respectively. This repartition did not differ significantly between Period1 and Period2 (P=0.27). Patients receiving insulin via any route were significantly more frequently in the 15-30% class (45.7% vs 25.1%; P<0.0001) or in the >30% class (21.3% vs 6.6%; P<0.0001) than patients not receiving insulin, as were patients with diabetes compared with patients without (15-30% class: 42.5% vs 32.9%, P=0.015; >30% class: 25.6% vs 9.7%; P<0.0001) (see **Fig.S9, Fig.S10 and Fig.S11**).

In the subset of patients receiving insulin of any type, glucose variability did not differ between patients receiving long-acting SC insulin (19.4% [IQR: 14.7; 30.2]) or not (19.4% [IQR: 13.2; 28.1]) (P=0.32). In this patient subset, the repartition of the individual glucose variability across the different classes (<15%, 15-30%, and >30%) did not differed significantly according to the existence of diabetes, Period1 or Period2, and the use of SC insulin (P=0.06) (**Fig.S12).**

**Fig.S9: Individual glucose variability according to the period (Period1 or Period2) and types of insulin used, in all patients (n=831*)**

*The individual glucose variability could be calculated for only 831 patients because 62 patients had zero or only one blood glucose value measured on the study day. The total of subgroup is > 831 because patients under IV + SC insulin are represented in each insulin subgroup.

Fig S9 caption: Pink points represent individual glucose variability (%) < 15%, purple points between 15 and 30%, red points > 30%. Boxplots represent the median and interquartile range of individual glucose variability. Percentages represent proportion of each glucose variability class.

**Fig. S10: Individual glucose variability according to the period (Period1 or Period2) and types of insulin used, in patients without diabetes (n=609*)**

*The individual glucose variability could be calculated for only 609 patients because some patients had zero or only one blood glucose value measured on the study day. The total of subgroup is > 609 because patients under IV + SC insulin are represented in each insulin subgroup.

Fig S10 caption: Pink points represent individual glucose variability (%) < 15%, purple points between 15 and 30%, red points > 30%. Boxplots represent the median and interquartile range of individual glucose variability. Percentages represent proportion of each glucose variability class.

**Fig. S11: Individual glucose variability according to the period (Period1 or Period2) and types of insulin used, in patients with diabetes (n=192*)**

*The individual glucose variability could be calculated for only 192 patients because some patients had zero or only one blood glucose value measured on the study day. The total of subgroup is > 192 because patients under IV + SC insulin are represented in each insulin subgroup. Percentages represent proportion of each glucose variability class.

Fig S11 caption: Pink points represent individual glucose variability (%) < 15%, purple points between 15 and 30%, red points > 30%. Boxplots represent the median and interquartile range of individual glucose variability.

**Fig. S12: Repartition of the individual glucose variability according to the period (Period1 or Period2), existence of diabetes, and use of subcutaneous insulin, in patients receiving insulin of any type (n=401)**

**Fig. S12 caption:**

In this mosaic plot, the numbers of patients belonging to each class of individual glucose variability (<15%, 15-30%, >30%) for each category (each combination of Period1 or Period2, diabetes or not, and subcutaneous insulin use or not) are superimposed on rectangles. The area of each rectangle is proportional to the number of patients it contains.

There was no significant difference in the repartition of patients across the different classes of individual glucose variability (G = 23.3, degrees of freedom = 14, P-value = 0.06). This analysis included only 401 patients upon the 408 patients receiving insulin of any type because individual glucose variability could not be calculated in 7 patients who had zero or only one blood glucose value measured on the study day.

**Fig. S13: Number of blood glucose tests performed according to the number of hypoglycaemias.**

Fig S13 caption: Pink points represent individual number of blood glucose tests performed on the study day according to the number of hypoglycaemias ordered from 0 to 4. Boxplots represent the median and interquartile range of the number of blood glucose test performed.

**Fig.S14: Number of blood glucose tests performed according to the number of hyperglycaemias.**

Fig S14 caption: Pink points represent individual number of blood glucose tests performed on the study day according to the number of hyperglycaemias ordered from 0 to 6. Boxplots represent the median and interquartile range of the number of blood glucose test performed.

**Table S6. Hyperglycemic events.**

|  | Overall | Period 1 | Period 2 | P-value**^a^** |
| --- | --- | --- | --- | --- |
| **Total No. of hyperglycemic events**  **(glycaemia >1.80 g/L)** *(upon total No. of blood glucose values reported)* | **1135 / 4823 (23.5%)** | **642 / 2594**  **(24.7%)** | **493 / 2229**  **(22.1%)** | **0.032** |
| In patients receiving insulin | 997 / 2670 (37.3%) | 538 / 1264 (42.6%) | 459 / 1406 (32.6%) | **<0.0001** |
| In patients not receiving insulin | 138 / 2153 (6.4%) **^b^** | 104 / 1330 (7.8%) **^b^** | 34 / 823 (4.1%) **^b^** | **0.0005** |
| In diabetic patients receiving insulin | 532 / 1148 (35.4%) | 264 / 505 (52.3%) | 268 / 643 (41.7%) | **0.0004** |
| In diabetic patients not receiving insulin | 13 / 120 (10.8%) **^b^** | 9 / 82 (11.0%) **^b^** | 4 / 38 (10.5%) **^b^** | **>0.99** |
| In non-diabetic patients receiving insulin | 458 / 1504 (30.4%) | 271 / 755 (35.9%) | 187 / 749 (25.0%) | **<0.0001** |
| In non-diabetic patients not receiving insulin | 125 / 2033 (6.1%) **^b^** | 95 / 1248 (7.6%) **^b^** | 30 / 785 (3.8%) **^b^** | **0.0004** |
| **No. of patients with at least one hyperglycemic event** | **402 / 893 (45.0%)** | **227 / 517 (43.9%)** | **175 / 376 (46.5%)** | 0.45 |
| In patients receiving insulin | 305 / 408 (74.8%) | 161 / 199 (80.9%) | 154 / 209 (73.7%) | 0.10 |
| In patients not receiving insulin | 87 / 485 (17.9%) **^b^** | 66 / 318 (20.8%) **^b^** | 21 / 167 (12.6%) **^b^** | **0.026** |
| In diabetic patients receiving insulin | 157 / 178 (88.2%) | 76 / 83 (91.6%) | 81 / 95 (85.3%) | 0.15 |
| In diabetic patients not receiving insulin | 9 / 34 (26.5%) **^b^** | 5 / 26 (19.2%) **^b^** | 4 / 8 (50.0%) **^c^** | 0.16 |
| In non-diabetic patients receiving insulin | 156 / 227 (68.7%) | 84 / 115 (73.0%) | 72 / 112 (64.3%) | 0.20 |
| In non-diabetic patients not receiving insulin | 78 / 451 (17.3%) **^b^** | 61 / 292 (20.9%) **^b^** | 17 / 159 (10.7%) **^b^** | **0.006** |

^a^: Proportions were compared by Fisher exact test

^b^: P<0.0001 when compared with patients who were receiving insulin

^c^: P=0.030 when compared with patients who were receiving insulin

**Table S7. Incidence rate ratio of hyperglycemia (whole population, n=893)**

|  | Incidence rate ratio  (95% confidence interval) | P value |
| --- | --- | --- |
| **Insulin use** | 6.20 (4.96; 7.75) | **< 0.0001** |
| Diabetes | 1.61 (1.35; 1.91) | **< 0.0001** |
| Period1 vs Period2 | 1.35 (1.13; 1.91) | **0.001** |
| SOFA (per 1-point increase) | 1.01 (0.99; 1.04) | 0.20 |
| Infection | 1.08 (0.90; 1.29) | 0.41 |
| Calories intake on the study day (per each kcal/kg of body weight increase) | 1.00 (1.00; 1.01) | 0.62 |
| Oral alimentation | 0.75 (0.59; 0.95) | **0.019** |

**Table S8. Incidence rate ratio of hyperglycemia (in patients receiving insulin of any type and route of administration, n=408)**

|  | Incidence rate ratio  (95% confidence interval) | P value |
| --- | --- | --- |
| **Use of subcutaneous insulin** | 3.45 (2.97; 4.00) | **<0.0001** |
| Diabetes | 2.15 (1.86; 2.48) | **<0.0001** |
| Period1 vs Period2 | 1.09 (0.93; 1.27) | 0.30 |
| SOFA (per 1-point increase) | 0.99 (0.97; 1.01) | 0.20 |
| Infection | 1.16 (0.99; 1.35) | 0.08 |
| Calories intake on the study day (per each kcal/kg of body weight) | 1.00 (1.00; 1.00) | 0.74 |
| Oral alimentation | 0.62 (0.50; 0.77) | **<0.0001** |

Incidence rate ratios were obtained by multivariable mixed-effect negative binomial regression with propensity score-based inverse probability of treatment weighting. Propensity score was the probability of being treated by subcutaneous insulin calculated through logistic regression using patient baseline characteristics as predictors.

**Table S9. Incidence rate ratio of hyperglycemia (in patients receiving insulin of any type and route of administration, n=408)**

|  | Incidence rate ratio  (95% confidence interval) | P value |
| --- | --- | --- |
| **Use of subcutaneous long-acting insulin** | 3.58 (2.84; 4.52) | **<0.0001** |
| Diabetes | 1.73 (1.48; 2.02) | **<0.0001** |
| Period1 vs Period2 | 1.23 (1.05; 1.45) | **0.013** |
| SOFA (per 1-point increase) | 1.02 (1.00; 1.04) | 0.051 |
| Infection | 0.99 (0.84; 1.17) | 0.92 |
| Calories intake on the study day (per each kcal/kg of body weight) | 1.00 (1.00; 1.01) | 0.42 |
| Oral alimentation | 0.90 (0.72; 1.13) | 0.38 |

Incidence rate ratios were obtained by multivariable mixed-effect negative binomial regression with propensity score-based inverse probability of treatment weighting. Propensity score was the probability of being treated by subcutaneous long-acting insulin calculated through logistic regression using patient baseline characteristics as predictors.

**References**

1. Zhang Z (2016) Multiple imputation with multivariate imputation by chained equation (MICE) package. Ann Transl Med 4:30. https://doi.org/10.3978/j.issn.2305-5839.2015.12.63

2. Austin PC, Stuart EA (2015) Moving towards best practice when using inverse probability of treatment weighting (IPTW) using the propensity score to estimate causal treatment effects in observational studies. Stat Med 34:3661–3679. https://doi.org/10.1002/sim.6607

3. Seaman SR, White IR (2013) Review of inverse probability weighting for dealing with missing data. Stat Methods Med Res 22:278–295. https://doi.org/10.1177/0962280210395740
